# A double-blinded randomized placebo-controlled non-inferiority trial protocol for postoperative infections associated with canine pyometra

**DOI:** 10.1186/s12917-023-03629-w

**Published:** 2023-06-20

**Authors:** Anna Ylhäinen, Sari Mölsä, Thomas Grönthal, Jouni Junnila, Merja Rantala, Outi Laitinen-Vapaavuori, Katariina Thomson

**Affiliations:** 1 Department of Equine and Small Animal Medicine, Faculty of Veterinary Medicine, University of Helsinki, Viikintie 49), P.O. Box 57, Helsinki, FI-00014 Finland; 2 Animal Health Diagnostics Unit, Finnish Food Authority, Mustialankatu 3, Helsinki, FI-00790 Finland; 3 EstiMates Oy, Tykistökatu 4, Turku, FI-20520 Finland; 4 Evidensia Eläinlääkäripalvelut Oy, Tammiston Kauppatie 29, Vantaa, FI-01510 Finland

**Keywords:** Pyometra, Dog, Antimicrobial resistance, Antimicrobial, Surgical site infection, Urinary tract infection, Randomized controlled trial

## Abstract

**Background:**

Pyometra is a common infectious condition, especially in elderly bitches. In addition to an infected uterus, dogs may have concurrent urinary tract infection (UTI). The preferred treatment is surgical removal of the ovaries and uterus, whereupon the general prognosis is excellent. In addition, antimicrobial therapy is frequently prescribed for postoperative treatment. However, no research exists on the benefit of postoperative antimicrobial treatment in uncomplicated canine pyometra. Antimicrobial resistance has become a major challenge in treatment of bacterial infections. Diminishing overuse of antimicrobial agents is essential for controlling the development of antimicrobial resistance in both animals and humans.

**Methods:**

This double-blinded, randomized, placebo-controlled two-arm clinical trial is designed to compare the incidence of postoperative infections associated with surgical treatment of uncomplicated pyometra followed by two different treatment protocols. For the study, 150 dogs presenting with an uncomplicated pyometra and that are to undergo surgical treatment will be recruited. Dogs with body weight < 3 or > 93 kg, complicated pyometra, primary disease increasing the risk of infection, or immunosuppressive medication will be excluded. All dogs will receive one dose of sulfadoxine-trimethoprim intravenously as an antimicrobial prophylaxis. Postoperatively, dogs will be randomized to receive either a five-day course of placebo or an active drug, sulfadiazine-trimethoprim orally. During the surgery microbiological samples will be taken from urine and uterine content. The follow-up includes a control visit in 12 days and an interview of the owner 30 days after surgery. If bacteriuria is detected at the time of surgery, a urinary sample will be cultured for bacterial growth at the control visit. The primary outcome is the incidence of a postoperative surgical site infection (SSI), and the secondary outcome is the occurrence of clinical UTI with bacteriuria. Intention-to-treat and per-protocol analyses will be performed to compare outcome incidences between the treatment groups.

**Discussion:**

Research-based evidence is necessary to create treatment guidelines for judicious use of antimicrobials. The goals of this study are to provide evidence for reducing the use of antimicrobials and targeting the treatment to patients proven to benefit from it. Publishing the trial protocol will increase transparency and promote open science practices.

**Supplementary Information:**

The online version contains supplementary material available at 10.1186/s12917-023-03629-w.

## Background

Antimicrobial resistance among bacteria is an increasing problem globally. Refraining from use of unnecessary antimicrobial medications and targeting the treatment to patients who clearly benefit from it are the main ways to control the development of antimicrobial resistance. In the European Union, all member countries are obligated to gather species-specific data on antimicrobial use [1]. In addition, several different organizations and government authorities have published guidelines for antimicrobial use in small animals [2–6]. Although many countries have guidelines on prudent use of antimicrobials, antimicrobial treatments are still often prescribed based on habit or fear of complications, not on evidence-based benefit to the patient [7]. Further, although a handful of studies have been published on protocols for shorter durations of antimicrobial treatment in small animals, randomized controlled trials (RCTs) on de-escalating or refraining from the use of antimicrobials are still largely lacking [8, 9]. Designing and carrying out RCTs are vital to evaluate which treatment protocols are of true benefit to patients.

Pyometra is a common condition, especially in elderly intact bitches. It is defined as an infection of the uterus in which pus accumulates within the lumen [10]. In a Swedish study based on insurance data, 24% of intact bitches aged under 10 years had suffered from pyometra [11]. Pyometra is a potentially fatal disease that can, if left untreated, rapidly develop to septicaemia due to spread of bacteria or their toxins to the bloodstream [12]. The uterine content may leak or the wall can rupture, with resulting peritonitis [13]. The diagnosis of pyometra is based on signalment and clinical signs, and the diagnosis is confirmed with diagnostic imaging and laboratory examinations [14, 15].

The most common infective agent in pyometra is *Escherichia coli* as an ascending infection through the vagina and cervix [16–18]. *E. coli* is a facultatively anaerobic Gram-negative enterobacterium that is a part of the normal intestinal microbiota in mammals [19]. Gram-negative bacteria produce endotoxins that can induce the cytokine cascade of the immune defence and the excretion of many inflammatory mediators, which may trigger the sudden inflammatory response syndrome (SIRS) [20, 21]. When SIRS is caused by an infection, as is the case in severe pyometra, the condition is defined as sepsis [22]. Left untreated, SIRS can develop into multi-organ dysfunction syndrome (MODS) and eventually death [22, 23].

The preferred treatment of pyometra is surgical removal of the ovaries and the infected uterus [24]. In general, the prognosis after a successful surgery is excellent, with death rates as low as 1% [13, 25]. In addition to peritonitis, the most common infectious complications associated with the condition include surgical site infection (SSI) and urinary tract infection (UTI) [13]. SSI may occur as superficial, deep or organ infections as described by Horan [26], the last-mentioned involving the uterine stump or abdominal cavity. SSI rates have rarely been reported after pyometra surgery [13], but the reported incidence of SSIs after clean-contaminated procedures varies between 3.5% and 6.6% [27–30]. UTI is a common disease, especially in bitches, due to their relatively short urethra [31]. It is caused by a temporary or permanent disturbance in the local defence mechanisms, which allows the attachment and multiplication of bacteria in the urinary tract [32]. In dogs with pyometra, UTIs have been reported in three studies, in which 5.6–71% of dogs were found to have concurrent cystitis. In two of these studies, the bacterial isolates were identical to the ones found in uteri [13, 16, 33]. However, no research data exist on the postoperative occurrence of UTI.

In addition to surgical removal of the infected uterus, the current recommendation is to administer antimicrobial prophylaxis [29, 34]. Some recent literature also recommends continuing antimicrobial administration for up to 14 days postoperatively [35]. In complicated pyometra, this can be justified [36]. However, no RCTs exist on the need or efficacy of the postoperative antimicrobial treatment in dogs with uncomplicated pyometra.

### Study objective

The objective here is to determine whether a difference exists in the incidence rate of postoperative infections associated with an uncomplicated pyometra surgery between dogs that receive antimicrobial prophylaxis only during the surgery (group A) and dogs that receive a 5-day course of antimicrobials postoperatively in addition to the prophylaxis during surgery (group B).

### Main hypothesis

The incidence of SSI associated with pyometra surgery is no higher than the predetermined non-inferiority margin in dogs in group A, relative to dogs in group B.

#### H_0_ (null hypothesis)

Placebo is inferior to antimicrobial treatment when assessing the incidence of infections associated with pyometra surgeries for 30 days postoperatively.

#### H_1_ (alternative hypothesis)

Placebo is non-inferior to antimicrobial treatment when assessing the incidence of infections associated with pyometra surgeries for 30 days postoperatively.

### Secondary hypothesis

The incidence of postoperative clinical UTIs with bacteriuria in a subpopulation of dogs with perioperative bacteriuria is no higher than the predetermined non-inferiority margin in dogs in group A, relative to dogs in group B.

### Sample size calculation

The sample size for the primary hypothesis is based on published literature and retrospective evaluation of the incidence of SSIs in dogs with pyometra at the Veterinary Teaching Hospital of the University of Helsinki (VTHUH). The SSI rate associated with the standard treatment protocol, antimicrobial prophylaxis during surgery followed by a 5-day course of trimethoprim-sulfonamide postoperatively, for uncomplicated pyometra at the VTHUH is approximately 1–2%, according to unpublished retrospective data from the two years prior to the start of this study. In the literature, the reported incidence of SSIs after clean-contaminated procedures is at most 6–7% [27–30]. Based on these infection rates, a difference (delta) of up to 7% in incidence is clinically acceptable and was chosen as the non-inferiority margin for the main hypothesis.

Table 1 shows a power analysis for sample size determination in different scenarios. A sample size of 150 dogs will provide adequate statistical power for the primary hypothesis, assuming the incidence of SSI in both groups is 1.3–2.7% (1–2/75) or the incidence is slightly higher in the placebo group (1.3% vs. 2.7%). The sample size has been determined for the primary hypothesis by utilizing a 5% two-sided type I error rate.

**Table 1:** Power calculations for the primary hypothesis

Power	Treatment, assumed %	Placebo, assumed %	Assumed difference in %	Non-inferiority margin	n/group
80%	1%	2%	1%	7%	51
80%	1%	3%	2%	7%	97
79%	1.33%	2.67%	1.33%	7%	75
47%	1.33%	4%	2.7%	7%	75
98%	1.33%	1.33%	0%	7%	75
84%	2.67%	2.67%	0%	7%	75
70%	4%	4%	0%	7%	75

The sample size determined above will also be sufficient to test the secondary hypothesis. Based on the literature, the proportion of dogs with concurrent bacteriuria varies widely [16, 33]. For our study, it was assumed that approximately 20% of dogs will have concurrent bacteriuria, and approximately 5% of them will develop a UTI postoperatively. The non-inferiority margin in the secondary hypothesis was set to 15%, based on the relatively low number of dogs with concurrent bacteriuria and the rare incidence of postoperative UTIs. Even though a 15% margin can be considered quite high for incidence of UTIs, it is widely used in clinical drug trials, and a difference of only a few infections between groups in a data set of this size signals an event against the hypothesis.

## Methods

### Study design

#### Study setting and source population

VTHUH is both a primary and referral hospital, with an annual caseload of approximately 18 000 companion animals, mainly dogs and cats. VTHUH sees approximately 70 cases of pyometra annually.

**Fig. 1:**
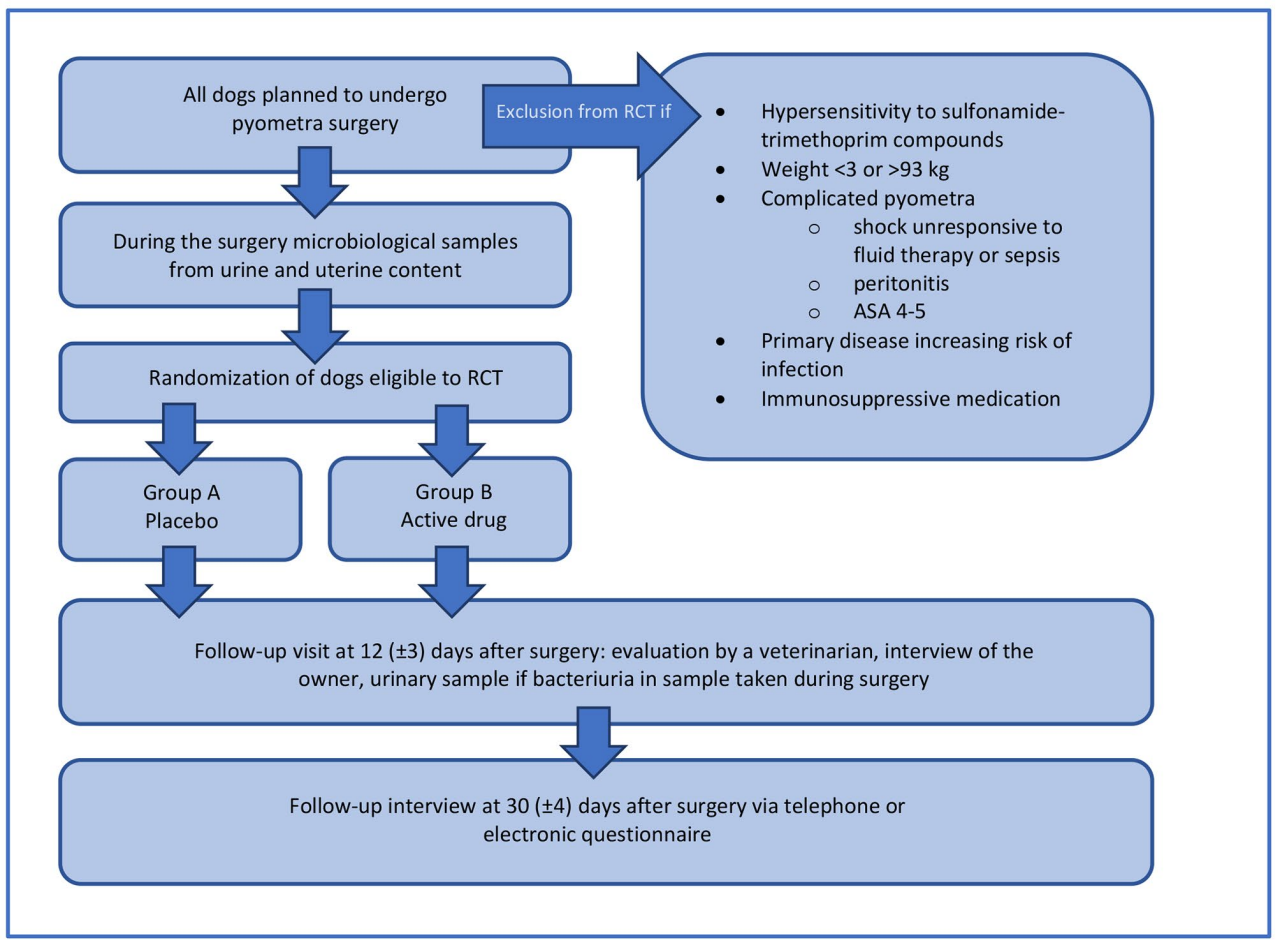
Study design for a two-arm non-inferiority randomized controlled trial (RCT) evaluating incidence rate of postoperative infections associated with an uncomplicated pyometra surgery. ASA: American Society of Anesthesiologists

All dogs presenting to VTHUH with pyometra that are to undergo surgical treatment will be eligible for the pyometra research project, which explores epidemiology and risk factors of SSIs and UTIs associated with pyometra surgery as well as bacteriology of these conditions. Owners of the dogs are informed about the project, and only dogs with their owner’s signed consent are included. As a part of this research project, a two-arm non-inferiority RCT will be conducted and is described here (Fig. 1). The RCT includes only VTHUH canine patients with uncomplicated pyometra. Although signed consent is requested from the owner prior to surgery, the eligibility of the patient to the RCT is confirmed during the pyometra surgery by the treating veterinarian.

Exclusion criteria are as follows:

- Body weight < 3 kg or > 93 kg
- Complicated pyometra
  - Shock unresponsive to fluid resuscitation or sepsis
  - Ruptured uterus and/or peritonitis
  - American Society of Anesthesiologists (ASA) physical status class 4–5
- Primary disease increasing risk of infection such as
  - Diabetes mellitus
  - Adrenal dysfunction
  - Chronic, severe liver or kidney disease
  - Malignant neoplasia
  - Urinary bladder neoplasia
  - Immunodeficiency
- Immunosuppressive medication
  - Prednisolone or prednisone > 1 mg/kg/d, or other glucocorticoids with equivalent dosage
  - Ciclosporin > 5 mg/kg/d
  - Azathioprine ≥ 2 mg/kg/d
  - Cytostatics at any dose
- Doberman Pinscher breed or known hypersensitivity to sulfonamide-trimethoprim compounds [37]

The patients excluded from the RCT can continue in other parts of the pyometra project.

#### Randomization

Eligible dogs to the RCT will be randomly assigned to receive either placebo (A) or an active drug (B) after the surgery using predetermined block randomization with blocks of four patients. Randomization is carried out in two strata according to patient weight (3–20 kg and 21–93 kg) to ensure optimal dosage of an active drug. The randomization list will be made by a statistician not involved in the statistical analyses of the study results. All personnel involved in treating the dogs or conducting the study as well as the owners of the dogs are blinded to the group assignment.

### Medications

#### Placebo and active control

All dogs will receive one dose of sulfadoxine-trimethoprim 30 mg/kg intravenously 30 min prior to skin incision as antimicrobial prophylaxis. After surgery, dogs will receive either placebo (group A) or sulfadiazine-trimethoprim (group B) 19 mg/kg BID orally for five days postoperatively. Study preparations are available in two formulations, 120 and 480 mg. Due to practical reasons in dosing, the final dosage may deviate +/- 3 mg/kg from the target dosage. The placebo will be manufactured using the same auxiliary substances as the active control (lactose, starch, microcrystalline cellulose, povidone, magnesium stearate and carmellose sodium), and the dosage of the tablets is identical.

The study preparations are packaged, and the placebo manufactured and packed at Töysä Pharmacy [38]. The packages are identical and are labelled with dosage instructions and randomization numbers. The packages are stored at the VTHUH and used in numerical order.

#### Other medications

Customary medications associated with the surgery or the comorbidities of the dog (such as analgesics, anaesthetics, fluids, antiemetics and other medications not mentioned in the exclusion criteria) are allowed. Glucocorticoids, ciclosporin or azathioprine are allowed if the dosage is below the immunosuppressive dose, as defined in the exclusion criteria. Medications for minor cardiac, pancreatic or thyroid insufficiency are also allowed. Vitamins, minerals or fatty acids used as a dietary supplement are allowed. Topical antimicrobial or corticosteroid treatments are allowed if they are not applied near the surgical site. Shampoos, including those containing chlorhexidine, are allowed.

### Course of the study and data recording

#### Primary visit

A veterinarian examines the dog as per usual according to the presenting complaint, including ultrasonographic evaluation of the uterus and assessment of blood values. After confirming the diagnosis of pyometra, the owner is informed about the study and written consent is requested. The owner’s informed consent includes a description of the study, the dog’s identification information and the owner’s name, signature and date. In addition, the owner fills out a questionnaire regarding the dog’s primary illnesses, medical history, all medications given within the previous week and current signs of illness. The veterinarian fills out a questionnaire regarding clinical examination findings, study number (running number starting from S-001) and randomization number (running number starting from 1001 or 2001, depending on body weight).

After abdominal incision, a urinary sample is obtained via cystocentesis and cultured onto CLED (Cystine Lactose Electrolyte Deficient, Thermo Scientific) or blood (trypticase soy agar with 5% sheep blood, Thermo Scientific) agar within 30 min. After removal of the uterus, a bacterial specimen of the uterine content is aspirated through the uterine wall and transferred into a transport medium (Portagerm, bioMerieux) or in case of a minimal amount of content, a small stab incision is made on the wall and a bacterial swab specimen is taken from the lumen of the uterus (M40 Transystem swabs, Copan). Uterine samples are cultured onto blood (as above), Chocolate + Vitox (Thermo Scientific), and Brilliance UTI (Oxoid) agars for aerobic or facultatively anaerobic species, and for FAA (fastidious anaerobe agar) with horse blood (Thermo Scientific), and Schaedler anaerobe KV selective (Thermo Scientific) agars for anaerobic species. Both aerobic and anaerobic cultures are performed. Bacterial isolates will be identified using matrix-assisted light desorption-ionization time-of-flight (MALDI-TOF) mass spectrometry, and susceptibility testing will be done by CLSI standardized disk-diffusion method, as described in the Finres-Vet report [39].

#### At home after surgery

The owners are instructed to monitor their dogs at home. In addition to general well-being, possible clinical signs of SSI, such as purulent drainage, redness, heat, local swelling or pain in the incision [26], and clinical signs of UTI, such as pollakiuria, stranguria, dysuria, hematuria or polydipsia/-uria [4], are monitored. Owners are instructed to contact VTHUH or the primary researcher if any signs develop.

#### Follow-up visit 12 d (± 3 d) after surgery

All dogs are evaluated by a veterinarian 12 days after surgery. A questionnaire is filled out with information about the physical examination, healing process, possible clinical signs of SSI [26] or UTI [4], other signs of illness and possible adverse reactions related to the study preparation. If bacteriuria was detected at the time of surgery and the dog has not been treated for a UTI during the follow-up period before the first visit, a urinary sample is taken via cystocentesis and cultured for bacterial growth. The sutures are removed if the incision was closed using non-absorbable suture material.

The owners are interviewed about whether the study preparation has been given as instructed. In addition, the packages with leftover tablets will be collected and the tablets counted by an assistant not involved in the treatment of dogs or the collecting of study data, recording the number of tablets returned compared with the number that should have been consumed during the treatment period according to the dog’s body weight (evaluation of owner compliance).

#### Follow-up interview 30 d (± 4 d) after surgery

The owners are interviewed via telephone or electronic questionnaire, and a questionnaire is filled out with information about possible clinical signs due to SSI or UTI, other signs of illness, veterinary appointments or medications during the 30-day follow-up period. In addition, possible protocol deviations are evaluated.

#### Electronic patient database and study database

Data relevant to patient care, monitoring, procedures, anaesthetic records and laboratory analyses are recorded to the electronic patient database (ProvetNet, Nordhealth Finland Oy) of VTHUH as usual.

Data from the electronic patient database are entered into an Epi-Info database (v. 7.2, CDC). The following data are recorded in the study database: research number, randomization number, dog’s demographic information, preoperative blood sample analysis (total leucocyte count, percentage of band neutrophils, thrombocyte count, lactate, glucose, albumin, total proteins, C-reactive protein), suture materials used in surgery, technique used for ligation of cervix and adjacent blood vessels, length of anaesthesia and surgery, time of antimicrobial prophylaxis, veterinary appointments and medications given during the follow-up period (30 d) as they are related to the outcomes, urine and uterine content culture results, and culture results from those infection sites that are related to the outcomes. No identification data apart from the patient number from the electronic patient database will be recorded in the study database. Both databases are used in accordance with General Data Protection Regulations (GDPR).

### Patient safety and adverse effects

Prior to the surgery, the owner of the dog is interviewed about any chronic diseases of the dog, current signs of illness and possible medications, allergies or hypersensitivities. A full clinical examination is performed and an abdominal ultrasound conducted to confirm the diagnosis of pyometra and to detect possible peritonitis. Vital signs and body temperature are monitored during anaesthesia. Recovery is monitored and the dog is discharged only after full recovery from anaesthesia and no longer requiring in-hospital care. Only dogs that are eligible based on primary evaluation, assessment of the severity of infection and the state of the uterus and peritoneum during surgery are selected for the study.

Sulfonamide-trimethoprim compounds have been reported to have the following side effects in dogs: polyarthritis, polyuria, vomiting, inappetence, diarrhoea, fever, thrombocytopenia, keratoconjunctivitis sicca and aplastic anaemia [40, 41]. Hepatitis, anaphylaxis and other hypersensitivity reactions are also possible [37]. Severe adverse effects associated with sulfonamide-trimethoprim medications are extremely rare when the treatment period is short, such as in this study [41]. The owners are instructed to monitor their dogs for possible adverse reactions to the medications and to contact VTHUH in such cases. In addition, possible adverse effects are queried at the follow-up interview and are recorded. If significant, life-threatening adverse effects are suspected, administration of the study preparation is ceased, and randomization is opened if deemed necessary. The ingredients used in the placebo are commonly used in manufacturing of tablets and are therefore not expected to have adverse effects.

During the study the incidences of infections associated with pyometra surgeries, especially severe complications such as peritonitis or stump pyometra, are assessed at regular intervals. If the incidences start to increase, the underlying reasons are investigated, and continuation of the study is reassessed.

### Discontinuation criteria and management

The owner of the dog may discontinue participation in the study at any point, without consequence. Time of discontinuation and the reason (if given) is added to the patient records. Information gathered until the time of discontinuation is used in the study.

If the clinical condition of the dog necessitates treatment that may alter the outcome of the study, or if the study preparation interferes with other necessary treatment, administration of the study preparation is discontinued, and the dog treated as necessary. Time of discontinuation of the study preparation and the reason for it is saved in the patient records. To ensure patient safety, the randomization may be opened for individual dogs. In this case, the information is added to the patient records.

### Outcome variables

#### Primary

Occurrence of SSIs within 30 days of surgery is considered the primary outcome variable (dependent variable). Any of the following are regarded as an SSI [26]:

- Superficial SSI (skin and/or subcutis only)
- Deep SSI (extending to linea alba)
- Organ infection: peritonitis and stump pyometra, and associated peritonitis

#### Secondary

Clinical UTIs with bacteriuria within 12 + 3 days of surgery in dogs having bacteriuria in the urinary sample taken during pyometra surgery is considered the secondary outcome variable [4].

### Protocol deviations

All protocol deviations and their significance to the outcomes are assessed individually, and all assessments are recorded. The parentheses indicate whether the deviation affects the primary or secondary outcome variable.

#### Minor

- Missing the follow-up visit, but healing of the incision can otherwise be reliably assessed (e.g. via video call or photographs).
- Not using an Elizabethan collar or the dog having access to lick the incision.
- Usage of wound care or antiseptic products after evidence of SSI.

#### Major

- Follow-up visit or final interview at a time remarkably outside the acceptable range and no other reliable way to assess SSIs or UTIs (primary and secondary outcome).
- If bacteriuria was detected at the time of surgery, the dog has not been treated for an UTI within 12 days of surgery and no follow-up visit is obtained (secondary outcome).
- Other abdominal surgery during the follow-up period that prevents assessment of the incision from the pyometra surgery (primary outcome).
- Treatment for UTI with systemic antimicrobials during the follow-up period for SSI (primary outcome).
- Treatment for SSI during the follow-up period for UTI (secondary outcome).
- Prohibited medications with assessment made individually based on the dosage and time of use.
  - Medications listed in the exclusion criteria, the dosage and time of use will be evaluated when assessing the effect of the deviation (primary and secondary outcome).
  - Systemic antibiotics for reasons other than those assessed in the study (primary and secondary outcome).
  - Owner-initiated local surgical site treatment without veterinary instruction such as honey-based or resin ointments, hypochlorous acid, polyhexamethylene biguanide (PHMB) or other local antiseptics (primary outcome).
  - Use of methenamine hippurate during the follow-up period for UTIs (secondary outcome).

### Statistical methods

#### Analytical populations (ITT, PP)

**Intention-to-treat (ITT)** population includes all randomized dogs that receive at least one dose of the blinded study preparation.

**Per-protocol (PP)** population is a subset of the ITT population, excluding dogs with major protocol deviations that can be considered to affect treatment results.

Both the ITT and PP populations are utilized in assessing the main hypothesis. To avoid increasing the rate of type I errors, the ITT population will be considered the main population. PP analyses will be done as robustness analysis to support the ITT analyses. Safety population is identical to the ITT population.

Dogs will be classified to analytical populations after the database has been locked and before opening of the treatment code for the blinded database.

#### General statistical notions

**The summary statistics** include the number of dogs, the mean, standard deviation, median, minimum and maximum for continuous variables, and the frequency and percentage for categorical variables. The results of the statistical tests (including 95% confidence intervals) will be presented where a formal analysis has been performed. In addition, line listings of all dogs will be provided.

**Multiplicity** correction will not be required, as the study has one main outcome variable and one treatment comparison, and the main analysis is based on the ITT population.

**Interim analyses** have not been planned. If the blinded assessments of the data during the study give reason for an interim analysis (e.g. a possible safety issue), one will be performed based on an independent decision and protocol.

**Missing data** associated with the main analysis are expected to be minor; therefore, missing data will not be imputed.

The main analysis will be conducted separately in at least the following **subsets**: concurrent bacteriuria detected at the time of surgery (yes/no) and size of dog used as a stratification factor (small/large).

**Statistical significance** will be interpreted as p-value < 0.05, unless otherwise stated.

#### Background variables

All background variables will be tabulated descriptively by treatment group. Patient disposition will be summarized.

#### Outcome variables

All outcome variables will be tabulated descriptively by treatment group.

##### **Primary** outcome variable

The incidence of SSIs within 30 days of surgery will be analysed mainly by the 95% confidence interval of the difference of the incidence of infections in the treatment groups. The upper limit of the confidence interval will be compared with a predetermined non-inferiority margin to establish non-inferiority. In addition, the main outcome variable will be analysed using logistic regression. The statistical model will include at least the treatment group as a fixed factor and, if possible, a stratification factor (dog size group). Odds ratio will be calculated alongside the 95% profile-plausibility confidence interval to quantify the difference between the groups. Based on VTHUH historical data (internal infection surveillance, unpublished data), the incidence of SSIs is rare. The rarity of the endpoints will be taken into account by applying Firth’s bias correction method, which maximizes the penalized plausibility function instead of the traditional maximum plausibility and gives results that are more reliable when the endpoints are rare [42].

##### Secondary outcome variable

The incidence of UTIs will be analysed with similar methods as the main outcome variable.

#### Safety variables

All safety variables are tabulated descriptively by treatment group. Safety variables include the number of discontinuations, reason for discontinuation, severe complications (death related or non-related to pyometra, or peritonitis or other infection necessitating another surgery during the follow-up period), possible adverse effects of the study preparation (polyarthritis, polyuria, emesis, inappetence, diarrhoea, fever, thrombocytopenia, keratoconjunctivitis sicca, hepatitis, anaphylaxis or other hypersensitivity reactions during the administration). Adverse reactions will be assessed for severity and correlation.

Study preparation compliance will be tabulated by treatment group.

#### Statistical software

Statistical analyses, tables, figures and patient listings will be prepared using SAS® version 9.4 or later (SAS Institute Inc., Cary, NC, USA).

### Quality assurance

#### Study personnel and training

All veterinarians and veterinary technicians employed at VTHUH participating in recruitment or treatment of study patients or collection of data will have written instructions, and information sessions will occur regularly regarding practical arrangements of the study, including samples collected for the study, questionnaire forms, instructions for dog owners and study preparations.

#### Biosafety

Infection site specimens and bacterial isolates are handled in a biosafety level 2 (BSL 2), with HEPA-filtered laminar-flow cabinets. Workers in the laboratory have extensive training and experience in handling bacteriological specimens and bacterial isolates.

#### Data control and monitoring

Research personnel will monitor the collection of all required data weekly. Data necessary for analyses are fed to the study database regularly. If data are missing, the dog owner will be contacted to obtain the missing information. If the missing data cannot be obtained, it will be recorded as missing. If a mistake is found in the data collection forms, the mistake will be crossed out and the correct information added. The revision is marked with the date and initials of the revising person, and the reason for revision noted. The forms will be kept in a locked location, accessible only by research personnel.

Data validity is checked before locking the database both computationally and manually. Data validation includes visual inspection and checking selected key data (date and time variables, weight, certain blood parameters) for logical consistency by programmed SAS scripts. After correcting discrepancies, a randomly selected sample of 10% of dogs is used for independent quality control by comparing the sample with the data in the electronic patient records and in collected paper forms. The accepted error rate will be 0% for critical variables (randomization number, outcome variables, presence of bacteriuria in surgery, patient demographics) and < 0.5% for other data. If the error rate exceeds the predefined rate, the process is iterated until an acceptable rate is attained. After validation, the database is locked to await statistical analysis.

### Ethical considerations

The study will be conducted following good clinical practices [43], regulations of the Finnish Medicines Agency Fimea [44] and the Finnish legislation.

The Finnish Medicines Agency has approved the study (Vetkl 04/2018). In addition, the Viikki Campus Research Ethics Committee of the University of Helsinki has reviewed the research plan, found the study to be ethically acceptable and approved the informed owner consent form (Statement 5/2018). The research plan was presented to the Project Authorization Board, which stated that there is no need for a permit because no additional procedures will be conducted on the animals than what is necessary for diagnosing and treating the disease.

The care of dogs participating in the clinical drug trial will only differ from standard care by administering the study preparation postoperatively. Otherwise, the dogs will receive standard treatment according to their illness. Throughout the study, the dogs are monitored for signs of infection.

Cystocentesis, which is collected from all dogs, is a common procedure when suspecting urinary disease. Commonly, the samples are collected from animals without sedation, but in this study the samples are collected during the surgery, while the animals are under general anaesthesia, and thus, no additional pain is caused by the procedure. The urinary samples collected at follow-up visits are obtained transabdominally from dogs that are awake, but this is justified to ensure clearing of the bacteriuria in dogs that had bacteriuria during surgery and to determine whether the dog needs treatment for the condition.

The incidence of SSIs and UTIs is not expected to be higher in dogs receiving placebo than in dogs receiving the active control. All surgeries are associated with a minor risk of SSI; our retrospective data shows approximately 1–2% of dogs developing an infection after pyometra surgery. Most of the infections are mild. A small proportion of the dogs may develop a severe infection that requires surgical treatment, such as stump pyometra or peritonitis, but these types of complications are generally associated with the dog’s primary illness or general condition. In our patient material, these types of infections occur rarely despite the dog receiving antimicrobials postoperatively. In this study, only dogs with no complicating factors are selected to the drug trial, and randomization is performed after the surgery to ensure patient safety. Concurrent UTIs have not been widely researched, but the assumed incidence of postoperative UTIs is low. The incidence of SSIs and UTIs is monitored regularly. If infection rates start to rise above the standard rates, the study protocol will be re-evaluated and the study discontinued if necessary.

## Discussion

We present a study protocol that aims to challenge the currently recommended antimicrobial treatment in cases of surgically treated uncomplicated pyometra. There is little or no evidence to support the current recommendation to continue antibiotic treatment for 10–14 days postoperatively. The recommendations are based on the assumption that dogs with pyometra often have a concurrent UTI. However, the research evidence to support this assumption is scarce [13, 16, 33]. In addition, it is not known whether surgical removal of the infected uterus also results in clearance of the bacteria from the urinary bladder. Further, recent evidence in both dogs and humans suggests subclinical bacteriuria does not require antimicrobial treatment [4, 45–49].

Sulfadoxine-trimethoprim was chosen as the treatment medication since it is recommended by Finnish national guidelines for both surgical prophylaxis and postoperative treatment of uncomplicated pyometra [50]. Sulfonamide-diaminepyrimidine combinations have bactericidal activity against a wide range of bacteria, including *E. coli* [51], the most commonly isolated bacteria in canine pyometra. National surveillance of *E. coli* isolates from Finnish dogs indicates that only a minor proportion (11–13%) of isolates are non-susceptible to trimethoprim-sulfamethoxazole [39]. For these reasons, we consider the selection of sulfadoxine-trimethoprim as justified.

In the field of veterinary medicine, RCTs remain relatively rare even though they are the epitome of medical research. Publishing the study protocol will increase transparency and promote open science practices. It may also decrease some of the effort needed for planning future studies, as the basic principles of this study are easily translated to other applications, which may then lead to more evidence-based standard practices and higher patient safety. The randomization and blinding procedures utilized in this study are designed to eliminate selection bias and bias due to data loss, which are common in veterinary studies [52–54].

Quality assurance is essential for well-designed studies, and this is taken into account in the present research protocol. However, there are always some factors that cannot be influenced like owner compliance. Clinical practice has shown that even with thorough instructions, some owners will still deviate from them significantly. We have attempted to solve this issue by including questions about the adherence to the protocol in multiple stages of the study and by checking the medication remaining after treatment. Still, we are dependent on the information given by owners. Compliance of the research personnel is also important. The dogs with pyometra are often treated as emergency patients and can be presented at all hours. While all personnel are trained to follow the study protocol, increased chance of human error is possible due to emergency patient load and late hours. Moreover, in all prospective studies with follow-up, there may be some drop-outs. A follow-up of 30 days, which is also used in this study, is a recommended follow-up period for surveillance of SSIs [26] and hopefully short enough to maintain owner compliance.

The main goal of this study is to provide evidence for reducing antimicrobial use in certain situations, although the use of antimicrobials is still warranted in many cases, especially in hospitalized patients. This study has significance for the health and well-being of both animals and humans because the results will offer a way to target the use of antimicrobials to dogs proven to benefit from them, at the same time reducing the use of unnecessary antimicrobials in dogs that do not. Research-based evidence is necessary to create treatment guidelines to ensure the efficacy of antimicrobials and to slow or even prevent the development of antimicrobial resistance.

## Electronic supplementary material

Below is the link to the electronic supplementary material.

Supplementary Material 1

## Data Availability

After publication, the anonymized data will be made available by the corresponding author on reasonable request for academic purposes. No identifying or confidential data will be shared.

## Abbreviations

ASA: American Society of Anesthesiologists
CLED: cystine lactose electrolyte deficient
GDPR: General Data Protection Regulations
ITT: intention-to-treat
MODS: multi-organ dysfunction syndrome
PHMB: polyhexamethylene biguanide
PP: per-protocol
RCT: randomized controlled trial
SIRS: systemic inflammatory response syndrome
SSI: surgical site infection
UTI: urinary tract infection
VTHUH: Veterinary Teaching Hospital of the University of Helsinki
